# Quantitative positron emission tomography reveals regional differences in aerobic glycolysis within the human brain

**DOI:** 10.1177/0271678X18767005

**Published:** 2018-03-23

**Authors:** Tyler Blazey, Abraham Z Snyder, Yi Su, Manu S Goyal, John J Lee, Andrei G Vlassenko, Ana Maria Arbeláez, Marcus E Raichle

**Affiliations:** 1 Mallinckrodt Institute of Radiology, School of Medicine, Washington University, St. Louis, MO, USA; 2 Department of Neurology, School of Medicine, Washington University, St. Louis, MO, USA; 3 Department of Pediatrics, School of Medicine, Washington University, St. Louis, MO, USA; 4 Department of Biomedical Engineering, Washington University, St. Louis, MO, USA

**Keywords:** Brain imaging, energy metabolism, glucose, metabolism, positron emission tomography

## Abstract

Glucose and oxygen metabolism are tightly coupled in the human brain, with the preponderance of the brain’s glucose supply used to generate ATP via oxidative phosphorylation. A fraction of glucose is consumed outside of oxidative phosphorylation despite the presence of sufficient oxygen to do so. We refer to this process as aerobic glycolysis. A recent positron emission tomography study reported that aerobic glycolysis is uniform within gray matter. Here, we analyze the same data and demonstrate robust regional differences in aerobic glycolysis within gray matter, a finding consistent with previously published data.

## Introduction

The energetic needs of the healthy human brain are almost entirely met by oxidative consumption of blood-borne glucose.^1,2^ However, a fraction of the brain's glucose uptake does not undergo oxidative phosphorylation. This effect conventionally is quantitated using the oxygen-glucose index (OGI), which is the molar ratio of oxygen to glucose consumption. If no alternative fuels are used and all glucose undergoes complete oxidative phosphorylation, the OGI is exactly 6. However, multiple studies have shown that the OGI of the young adult human brain is less than 6, typically on the order of 5.5.^3–7^ Thus, around 10% of the whole brain’s glucose consumption is metabolized through non-oxidative pathways. We define aerobic glycolysis (AG) as the fraction of glucose metabolized outside of oxidative phosphorylation. AG is defined inversely proportional to OGI; thus, areas of the brain that have high AG have low OGI ratios and vice versa.

Prior work from our laboratory has shown that, in resting, healthy young adults, AG is regionally greater in prefrontal cortex, lateral parietal lobe, and the precuneus/posterior cingulate cortex, relative to the rest of the brain.^8^ These regions correspond to the default mode and fronto-parietal control (FPC) networks, which are areas of the cerebral cortex associated with higher-order cognition.^9^ Conversely, AG in the cerebellum has been shown by us,^8^ and others,^10^ to be lower than in the rest of the brain. Hyder et al.^7^ recently published a study disputing the existence of regional variability in AG. Using quantitative positron emission tomography (PET) techniques, Hyder et al. measured OGI in 13 normal volunteers and reported that OGI is uniform within gray matter, which implies that AG is uniform as well. In the following, we refer to this study as “Hyder et al.” To resolve the discrepancy between Hyder et al. and our previous findings, we reanalyzed the PET data from Hyder et al., which was generously shared with us by the original authors.

## Methods

### Dataset

We obtained processed, quantitative PET images of cerebral blood flow (CBF), oxygen utilization (CMRO_2_), and glucose consumption (CMRGlc) for 13 normal adult males from Hyder et al.^7^ CBF and CMRO_2_ were measured using [^15^O]H_2_O and [^15^O]O_2_, respectively. A two-compartment (tissue and vascular distribution) kinetic model was used for both tracers.^11,12^ No correction for recirculating [^15^O]H_2_O was performed during [^15^O]O_2_ modeling. CMRGlc was obtained by fitting an irreversible two-compartment (free [^18^F]FDG and trapped [^18^F]FDG-6-phosophate) model to the [^18^F]FDG data.^13^ No correction for vascular radioactivity was performed, and a lumped constant of 0.8 was used. All PET imaging data were acquired with arterial sampling, allowing for absolute quantitation of all metabolic parameters. For further methodological details, please see the original publication.^7^ As stated in the original report by Hyder et al.,^7^ all subjects gave written informed consent in accordance with the Helsinki Protocol and all experimental procedures were approved by the ethical review committees of the Central Denmark Region and the Aarhus University Hospital, Aarhus Denmark.

### OGI regional computations

To assess regional differences in AG, we first calculated voxelwise OGI (CMRO_2_/CMRGlc) in each subject. We then computed regional average OGI values in several regions of interest (ROI). Prior to computing regional means, we excluded voxels that were outside five median absolute deviations (1.11) from the gray matter median (4.83).^14^ Excluded voxels were predominantly in areas of vascular artifact or on the edges of the PET images (4.09% of all voxels were excluded). We also excluded any voxels that were not classified as gray matter in the atlas used by Hyder et al.^7^

**Figure 1.**
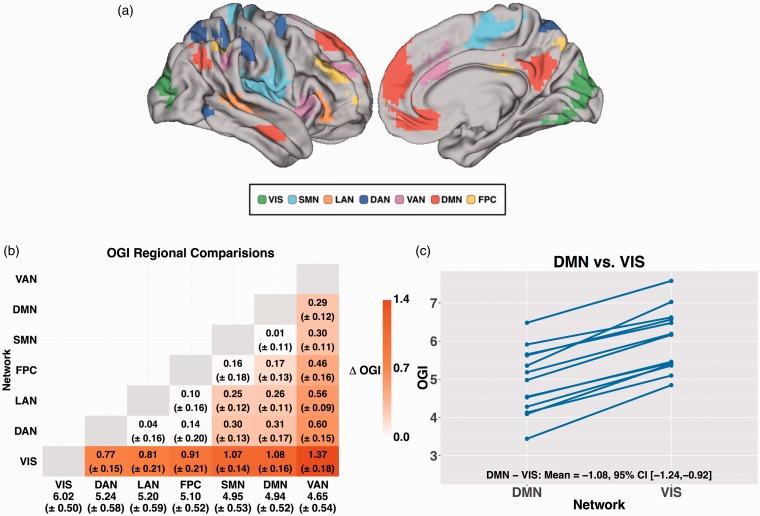
Differences in OGI between resting state networks. (a) Regions of interest for each of the seven resting state networks projected on the right hemisphere cortical gray matter surface of the Conte 69 atlas^41^ using Connectome Workbench.^42^ Images show the right lateral and medial surfaces. (b) Pairwise differences between each resting state network. Within each cell is the difference in OGI (ΔOGI) between resting state networks along with the 95% CI of the difference. Positive numbers indicate greater OGI (less AG) in the network listed on the vertical axis. Only significant differences are shown in color. The numbers along the bottom row are the mean and the 95% CI for each network. Network abbreviations: FPC: fronto-parietal control, DMN: default mode, DAN: dorsal attention, VAN: ventral attention; LAN: language; SMN: somatomotor; VIS: visual. (c) Within-subject comparison of OGI evaluated within the default mode network versus visual network. The solid blue lines connect regional measurements within a single participant. Note consistency of regional differences in OGI from subject to subject. The DMN exhibited lower OGI than the visual network (VIS) in every subject.

Our primary ROI set comprised seven resting state networks (Figure 1(a)), defined in a previous resting-state functional magnetic resonance imaging study.^15^ Each ROI included only voxels in which the likelihood of network identity exceeded 90%. Resting state ROIs were transformed, using FSL,^16,17^ into the atlas space used by Hyder et al. without alterations of the metabolic imaging data. We also created an ROI of the cerebellar gray matter within the atlas used by Hyder et al.^7^ To accommodate incomplete cerebellar coverage of the PET data, the present results are limited to portions of the cerebellum in which the OGI was measured in every subject (Supplementary Figure 1).

### Statistical methods

All statistical analyses were conducted in R.^18^ A one-way ANOVA with region as a factor and subject as a repeated measure was used to determine if brain region explained any variance in OGI. Statistical significance was determined using an *F-*test on the region factor. One sample *t-*tests were used to determine if regional OGI values were different from 6. An OGI significantly (*p* < 0.05, two-tailed) less than 6 means that the probability of finding such, or more extreme, data by chance is below 5%. We took this as indication that a portion of the glucose consumption in a given region undergoes only AG. In the same sense, paired *t*-tests were used to assess differences in OGI between regions. We used a significant difference (*p* < 0.05, two-tailed) as indication that AG is different between two regions. Correction for the 21 pairwise comparisons between networks was performed using false discovery rate (FDR) theory.^19^ Reported values are means and 95% confidence intervals unless otherwise stated.

The statistical thresholds that we defined above are dependent on the power of the Hyder et al. dataset. To determine the power of the Hyder et al. data, we performed a power analysis using two previously published PET datasets. All power calculations were performed using the R package pwr.^20^ Sasaki et al. reported the mean difference between the cortical and cerebellar gray matter OGI to be −1.48 (SD = 0.42; *n* = 7).^10^ The 13 subjects in the Hyder et al. dataset give us 100% power to detect an effect of this magnitude. The mean OGI difference between the cortical gray matter and the basal ganglia was found by Hatazwa et al. to be 0.38 (SD = 0.93; *n* = 7).^21^ The Hyder et al. dataset would provide only 17.2% power to detect this effect. Taken together, these analyses reveal that we are more than sufficiently powered to detect large regional differences, but are unlikely to capture smaller effects.

## Results

### AG varies by resting state network

To assess regional differences in AG, we computed OGI in seven resting state network (Figure 1(a)). The means for other metabolic parameters (e.g. CBF) are reported in Supplemental Table 1. With the exception of the visual network (VIS), all resting state networks had an OGI significantly less than 6 (*p* < 0.05), indicating the presence of AG. A repeated measures, one-way ANOVA revealed a highly significant difference in OGI across the brain (*F*_6,72_ = 74.16, *p* < 0.001). Differences in OGI between specific network pairs are shown in Figure 1(b); the RSNs are ordered by OGI and significant differences (*p* < 0.05, corrected) are highlighted by color. In agreement with previous work,^8^ the OGI was low in default mode network (DMN) and high in the visual network (VIS). Unexpectedly, the ventral attention (VAN) network had the lowest OGI. We note that these regional differences were highly consistent. For example, OGI in the DMN was less than OGI in the visual network (VIS) in every subject (Figure 1(c)).

### AG in the cerebellum

Previous studies have shown that AG in the cerebellum is lower than that in the rest of the brain.^8,10^ In the Hyder et al. data, the OGI in the superior cerebellum (see Methods) was 6.50 (±0.67), which was not significantly different from 6.0 (*t* = 1.63, *p = *0.13). The difference between the cerebellum and the rest of gray matter (5.18 ± 0.51) was significant (*t* = −8.70, *p* < 0.001). As the lumped constant in the cerebellum has been reported to be approximately 1.14 times greater than in the whole brain,^22^ we repeated our analysis after adjusting the cerebellum OGI for this difference. After the adjustment, the cerebellar OGI was 5.70 (±0.58), again not significantly different from 6.0 (*t* = −1.12, *p* = 0.28), but still significantly different from the rest of gray matter (*t* = −4.00, *p* = 0.0018). Thus, the cerebellum is characterized by a distinct lack of AG.

### Topography of OGI

**Figure 2.**
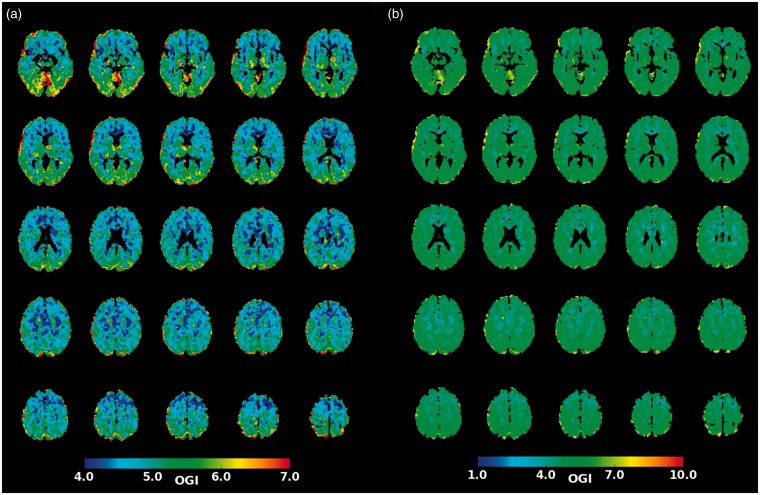
Regional topography of OGI. (a) A group-averaged OGI map obtained from the authors of the Hyder et al. study. Regional differences are found throughout the brain. (b) Replication of Figure 3(a) from Hyder et al.^7^ shows little regional variation in OGI. Regional differences are masked by the use of a color scale that lacks a dynamic range which is not matched over the relevant physiologic range of the data.

The present results indicate that regional differences in AG exist between resting state networks as well as between the cerebellar and non-cerebellar gray matter. Figure 2(a) shows group averaged OGI (image obtained from the original authors) at a finer spatial scale. This figure is essentially identical to Figure 3(a) in Hyder et al. (reproduced here as Figure 2(b)) except for choice of color scale. Thus, presenting the identical results using a more physiologically meaningful scale (4–7 in Figure 2(a) as opposed to 1–10 in Figure 2(b)) demonstrates regional differences in OGI on inspection.

## Discussion

Our reexamination of the data from Hyder et al. reveals two primary findings. First, many regions of the brain exhibit AG at rest. This result is consistent with both the regional PET literature^10,21^ as well as with whole-brain measurements of OGI.^3–6^ Second, we observed significant regional differences in AG between gray matter regions that were highly preserved across subjects (Figure 1(c)).

These findings are consistent with Vaishnavi et al.,^8^ a previous study from our group that employed regional standardized uptake ratios. The principal result of that study was that AG is significantly non-uniform across the brain. In particular, regions constituting the default mode network (DMN) had higher AG than other parts of the brain. In contrast, the cerebellum had lower AG. These findings are replicated here using the Hyder et al. dataset. There are, however, a few differences between the two datasets. The FPC network had higher AG in the Vaishnavi et al. study compared to Hyder et al., and the AG in the ventral attention network (VAN) was much higher in the Hyder et al. data compared to Vaishnavi et al. (Figure 1(b) and (c)). It is not clear whether these differences are attributable to analytical approach (relative vs. quantitative PET), study population (the Hyder et al. study contained only male subjects), or other unknown factors. Therefore, although both datasets clearly support regional differences, more work is needed to resolve the discrepancies between the two studies.

On the basis of the same dataset, Hyder et al. argued that no regional differences in AG exist, and that findings reported by Vaishnavi et al. are artifacts attributable to the use of relative metabolic measures. The present results, obtained using the quantitative data identical to that from the Hyder et al. study, do not support this perspective. It follows that the discrepant perspectives are attributable to difference in analysis methodology. Specifically, Hyder et al. did not account for subject level variability common to all regions (e.g. use of ANOVA without a repeated measures factor). Figure 1(c) illustrates how OGI measures in two regions would appear to be not significantly different if variability attributable to subject is not taken into account.

Could observed regional difference arise from non-biological artifact? PET involves many technical decisions including choosing a kinetic model, accounting for vascular radioactivity, adjusting for recirculating metabolites, and correcting for the delay and dispersion of the arterial input function. Any of these factors could, in theory, produce an artefactual regional difference in AG. However, we think this unlikely for several reasons. First, despite the fact that there are regional differences in cerebral blood volume^23^ and arterial delay,^24^ there is no direct evidence that any of these technical factors produce a spatial artifact that induces regional differences in OGI. Second, using different procedures to analyze PET data, we^8^ and others^10^ have found regional differences in OGI similar to the present findings. Finally, additional evidence from different techniques suggests that AG varies throughout the brain. Using microdialysis in a transgenic mouse model of Alzheimer’s disease (AD), Bero et al.^25^ reported regional differences in lactate levels in interstitial fluid, a result consistent with regional differences in AG. Taken together, the available evidence supports the conclusion that regional differences in OGI are of biological origin.

In the Hyder et al. dataset, AG accounts for 5.57 (±2.65) µMol/hg/min, or approximately 19%, of the glucose consumption in the DMN. From an energetic perspective, it may be surprising that AG accounts for so much glucose consumption in any part of the brain, as the quantity of ATP generated by AG is quite small compared to that generated by oxidative phosphorylation.^7^ Therefore, a number of alternative explanations have been proposed, including rapid synthesis of ATP for the Na^+^/K^+^-ATPase,^26^ generation of biosynthetic intermediates necessary for myelination as well as synaptic and neuritic formation and turnover,^8^ alteration of cellular redox potentials,^27^ regulation of glycogen levels through a hypothesized glycogen shunt,^28^ and the uptake and recycling of glutamate by astrocytes.^29,30^ The exact apportionment of AG among these alternatives remains uncertain.

One way to elucidate the role of AG in the brain is through spatial topography. Past work in our laboratory has shown that the spatial distribution of AG correlates with the expression of genes related to synaptic development and growth.^31^ The relationship between AG and synaptic plasticity is particularly intriguing given previous findings relating AG to task performance. Madsen et al.^32^ found that whole brain AG was elevated both during and after performance of the Wisconsin Card Sorting Test. Our group recently expanded on this finding. We measured regional OGI in subjects before and after the performance of a covert motor learning task.^33^ We found that hours after the performance of the learning task, subjects had elevated AG in the left Brodmann area 44, an area recruited by task performance. Furthermore, we observed a correlation between task performance and subsequent increases in AG. These results link focal changes in AG to learning and suggest that regional differences in AG might reflect regional differences in synaptic plasticity.

Other experiments have focused on the role of AG in aging and AD. For example, it has been shown that higher levels of neural activity lead to increased amyloid-beta production in a mouse model of AD.^25^ Moreover, this effect is associated with increased lactate levels in the interstitial fluid.^25^ Cross-sectional studies in humans have found that brain AG decreases in AD^34,35^ as well as in normal aging^36^ (two smaller aging studies have reported non-significant trends^37,38^). One interpretation of these findings is that the same processes that lead to high AG and synaptic plasticity in early life may ultimately lead to disease later in life.^39,40^

Synaptic plasticity is but one of several, non-exclusive explanations for the brain’s use of AG. Much more work is needed before AG in the brain is fully understood.^43^ Any explanation of AG will need to consider regional differences, which have now been reproduced in an independent dataset. It is our hope that this report will serve as an impetus for new research that will further elucidate the role AG in the brain.

## Supplemental Material

Supplemental material for Quantitative positron emission tomography reveals regional differences in aerobic glycolysis within the human brain Click here for additional data file. Supplemental material for Quantitative positron emission tomography reveals regional differences in aerobic glycolysis within the human brain by Tyler Blazey, Abraham Z Snyder, Yi Su, Manu S Goyal, John J Lee, Andrei G Vlassenko, Ana Maria Arbeláez and Marcus E Raichle in Journal of Cerebral Blood Flow & Metabolism
