# Supplementary material for: Quantitative positron emission tomography reveals regional differences in aerobic glycolysis within the human brain
Source: J Cereb Blood Flow Metab. 2018 Mar 23;39(10):2096–102. doi: 10.1177/0271678X18767005 (PMC6775584; doi:10.1177/0271678X18767005)
Supplement: Supplemental material for Quantitative positron emission tomography reveals regional differences in aerobic glycolysis within the human brain [file Supplemental_material.pdf]

Supplemental Material for Blazey et al., 2017

Supplemental Figure 1: Cerebellar gray matter region of interest shown in the space of the PET data. Region was derived from the atlas used by Hyder et al.[1]. Only voxels for which OGI was non-zero in every subject were included. The MNI152 T1 template was resampled to the space of the PET data for anatomical reference.

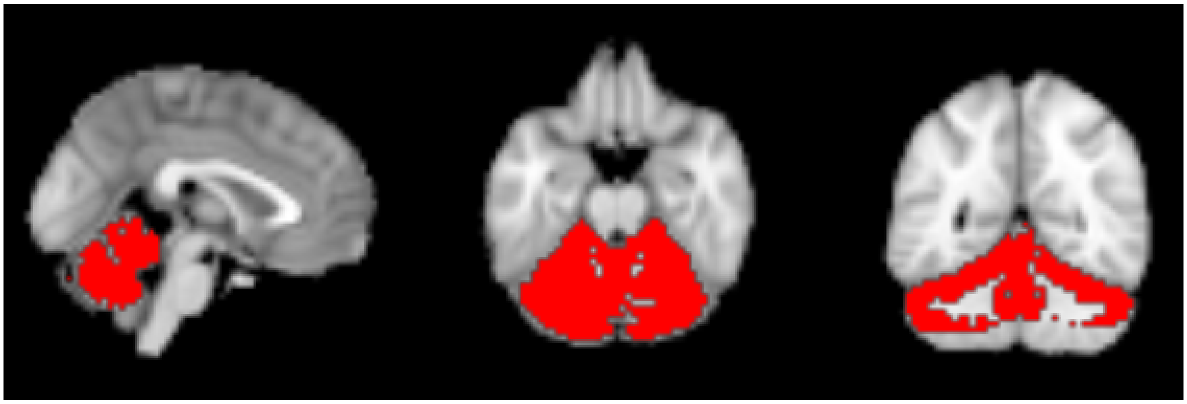

Supplemental Table 1: Means and 95% CIs for selected regions and resting state networks. Whole brain, gray matter, and white matter regions were taken from the atlas used by Hyder et al.[1]. Resting state regions were from Hacker et al.[2]. The cerebellum was excluded from all regions. Summary statistics were calculated after excluding voxels that exceed five median absolute deviations from the gray matter median[3]. The reported means for whole brain, as well as gray and white matter, are largely similar to those reported in the Hyder et al. manuscript[1].

Region	CBF (mL/hg/min)	CMRglc (μMol/hg/min)	$CMRO_2$ (μMol/hg/min)	OGI	OEF
**Whole Brain**	36.32 (2.89)	26.34 (1.59)	134.53 (13.76)	5.10 (0.50)	0.42 (0.04)
**Gray Matter**	38.31 (3.14)	27.57 (1.74)	142.51 (14.89)	5.18 (0.51)	0.42 (0.04)
**White Matter**	28.95 (2.11)	21.74 (1.15)	104.93 (9.79)	4.78 (0.47)	0.41 (0.04)
**Dorsal Attention**	37.97 (3.90)	29.00 (1.98)	150.32 (17.26)	5.24 (0.58)	0.44 (0.05)
**Ventral Attention**	45.40 (4.02)	32.13 (2.10)	147.13 (16.83)	4.65 (0.54)	0.37 (0.04)
**Somatomotor**	39.51 (3.42)	28.99 (1.81)	141.74 (16.59)	4.95 (0.53)	0.41 (0.05)
**Visual**	41.06 (3.35)	28.42 (2.21)	173.26 (17.46)	6.02 (0.50)	0.47 (0.05)
**Fronto-Parietal Control**	40.63 (3.81)	30.17 (2.08)	152.25 (16.21)	5.10 (0.52)	0.42 (0.05)
**Language**	41.58 (3.73)	29.88 (1.81)	152.86 (16.73)	5.20 (0.59)	0.41 (0.05)
**Default Mode**	41.03 (3.57)	29.67 (1.81)	144.73 (15.40)	4.94 (0.52)	0.40 (0.04)

References:

1 Hyder F, Herman P, Bailey CJ, Møller A, Globinsky R, Fulbright RK *et al.* Uniform distributions of glucose oxidation and oxygen extraction in gray matter of normal human brain: No evidence of regional differences of aerobic glycolysis. *J Cereb Blood Flow Metab* 2016; **36**: 903–916.

2 Hacker CD, Laumann TO, Szrama NP, Baldassarre A, Snyder AZ, Leuthardt EC *et al.* Resting state network estimation in individual subjects. *Neuroimage* 2013; **82**: 616–633.

3 Leys C, Ley C, Klein O, Bernard P, Licata L. Detecting outliers: Do not use standard deviation around the mean, use absolute deviation around the median. *J Exp Soc Psychol* 2013; **49**: 764–766.
